# Therapeutic Efficacy of Selenium Pre-treatment in Mitigating Cadmium-Induced Cardiotoxicity in Zebrafish (Danio rerio)

**DOI:** 10.21203/rs.3.rs-4583781/v1

**Published:** 2024-07-03

**Authors:** Rachael M. Heuer, Priscila Falagan-Lotsch, Jessica Okutsu, Madison Deperalto, Rebekka R. Koop, Olaedo G. Umeh, Gabriella A. Guevara, Md Imran Noor, Myles A. Covington, Delia S. Shelton

**Affiliations:** University of Miami; Auburn University; University of Miami; University of Miami; University of Miami; University of Miami; University of Miami; University of Miami; University of Miami; University of Miami

**Keywords:** Cadmium, Selenium, heart rate, epicardial edemas, zebrafish

## Abstract

Cardiovascular diseases are a rampant public health threat. Environmental contaminants, such as Cadmium (Cd), a toxic metal, have been linked to increased risk for cardiovascular diseases. Given that human exposure to Cd is increasing overtime, there is a need to develop new therapies to ameliorate Cd toxicity. Selenium (Se), an essential trace element, has been proposed to rescue the effects of Cd toxicity, with mixed effects. Se’s narrow therapeutic window necessitates precise dosing to avoid toxicity. Here, we assessed the effects of various waterborne Cd and Se concentrations and sequences on cardiac function using zebrafish (*Danio rerio*). We showed that Cd induced pericardial edemas and modified heart rates in a concentration-dependent manner. To identify the therapeutic range of Se for Cd-induced cardiotoxicity, zebrafish embryos were treated with 0, 10, 50, 100, 150, or 200 μg/L Se for 1–4 days prior to exposure to Cd at 2.5, and 5 μg/L. We found that a 50 μg/L Se pre-treatment prior to Cd at 2.5 μg/L, but not at 5 μg/L, reduced the prevalence of pericardial edemas and ameliorated Cd-induced bradycardia in zebrafish. Embryos exposed to 10 and 50 μg/L of Se showed typical heart morphology, whereas other Se-exposed and Se-deficient fish presented pericardial edemas. Longer Se pre-treatment durations led to fewer incidences of pericardial edemas. Overall, this study highlights the importance of optimizing Se concentration and pre-treatment periods to harness its protective effects against Cd-induced cardiotoxicity. These findings provide insights into potential therapeutic strategies for reducing Cd-related cardiovascular damage in humans.

## Introduction

Cardiovascular diseases (CVDs) have long been a critical public health issue and remains a leading cause of death worldwide (Timmis et al., 2023).According to the World Health Organization (WHO), CVDs account for an estimated 17.9 million deaths globally each year (Kaptoge et al., 2019). CVDs encompass a group of disorders affecting the heart and blood vessels, including coronary heart disease, heart failure, heart rate variability, and stroke (ref). Established risk factors for CVDs include age, sex, family history, and lifestyle factors such as tobacco use, unhealthy diet, obesity, physical inactivity, stress, and alcohol consumption (CDC, 2024; Flora & Nayak, 2019). In recent years, exposure to environmental pollutants, such as fine particulate matter (PM2.5) in the air and toxic metals like cadmium, lead, and arsenic, has been strongly linked to the progression of CVDs and the incidence of cardiovascular events (Hamed, 2022; Sun, 2023). In 2019, nearly 62% of all deaths related to environmental pollution were attributed to CVDs, according to the Global Burden of Disease research (Murray et al., 2020). While air pollution is already recognized as a risk factor for CVDs, medical societies have not yet uniformly addressed vascular toxicity from contaminant metals despite epidemiological evidence linking chronic exposure to low and low-moderate levels of these metals (Lamas et al., 2023).

Cadmium (Cd) is a trace metal present in the natural environment in rocks and soil, and is released into water and air during weathering processes(Fuller et al., 2022; Landrigan et al., 2018; Satarug & Phelps, 2020). However, anthropogenic activities such as mining and other industrial processes release Cd into the environment at concentrations toxic to both humans and ecosystems (Françoise Pinot et al., 2000). Cd was recently ranked seventh on the Agency for Toxic Substances and Disease Registry substance priority list, underscoring the need to better understand its impacts on human health (Agency for Toxic Substances and Disease Registry, 2022). Routes of exposure in occupational settings include the extraction and processing of ore, processing of Cd-containing industrial waste, or through phosphate fertilizers (Françoise Pinot et al., 2000; Thun et al., 1991; Verbeeck et al., 2020). Non-occupational exposure can occur through cigarette smoking, or exposure through Cd-contaminated water or air (Satarug & Moore, 2004). However, the primary source of exposure tends to be from diet (Jean et al., 2018; Wang et al., 2021). Of particular concern is Cd’s long half-life, which can span decades and lead to various human health impacts (Satarug et al., 2010).

The negative health impacts of Cd exposure in humans, including genotoxic damage, nephrotoxic injury, disruptions to calcium homeostasis, osteomalacia (Palus et al., 2003; Satarug & Moore, 2004; Staessen et al., 1996), and its classification as a known carcinogen (Hartwig, 2013), have been well-documented. Cd is known to target multiple organ systems and could predispose humans to increased risk of hypertension, Alzheimer’s disease, and impaired reproductive health (Kumar & Sharma, 2019; Satarug & Phelps, 2020). Increasing attention has focused on the impacts of Cd on the cardiovascular system. A recent population-based study reported a significant association between the blood levels of Cd and increased susceptibility to coronary heart disease (Ci et al., 2024), hypertension (Shin et al., 2012), and arterial disease (Tinkov et al., 2018). While the association between Cd and cardiovascular disorders is becoming more established, the pathophysiological mechanisms require further (Lamas et al., 2023).

Zebrafish serve as a well-established model for investigating how environmental pollutants, including metals, impact development, morphology and cardiovascular system in the lab (Chen et al., 2021; Shelton et al., 2023, 2024; Staudt & Stainier, 2012). Field studies of zebrafish help us ground our metrics ethologically (Kelly et al., 2021; Shelton et al., 2020; Suriyampola et al., 2016). Cellular differentiation and cellular migration during development, and the electrical properties of zebrafish hearts, closely mirror patterns observed in mammals, making them valuable for studying physiological and molecular mechanisms underlying toxicant exposures (Heideman et al., 2005; Staudt & Stainier, 2012). Their transparent early life stages allow for easy visualization of basic cardiovascular responses, including cardiac output (consisting of stroke volume and heart rate), the presence of pericardial edemas, and heart malformations (Perrichon et al., 2017). Various transgenic zebrafish lines enable visualization of cardiac malformations following toxicant exposures in live animals (Heideman et al., 2005). Furthermore, unlike mammals, zebrafish are capable of regenerating cardiac tissue following injury, providing valuable insights into the molecular mechanisms underlying cardiac regeneration in vertebrate tissues (Lepilina et al., 2006; Poss et al., 2002) and how toxicants can impact these repair processes (Hofsteen et al., 2013).

Cd is known to cause a wide array of impacts in larval and adult zebrafish (Avallone et al., 2015; Shelton et al., 2023, 2024; Wold et al., 2017). Impairments to the cardiovascular system vary across studies and are both concentration- and life-stage specific. Common themes are that Cd often leads to cardiac edema (Cheng et al., 2000; Mitovic et al., 2021; Yin et al., 2014)) and/or reduced heart rate (bradycardia) (P. Liu, Zhao, et al., 2021). For example, in developing zebrafish, waterborne exposure to 1.0 μM Cd led to a reduced heart rate at 72 Hours Post-Fertilization (hpf), but not at 48 hpf (P. Liu, Wang, et al., 2021). Another study, testing a wider concentration response through 4 Days Post-Fertilization (dpf; 0.01–10 μM, 1.124–1124 μg/L waterborne Cd), showed tachycardia (increased heart rate) at 5 dpf. However, when these same groups were later examined as adults after being reared in control water (8–10 months), zebrafish exposed to Cd during development exhibited an inconsistent concentration response, where bradycardia was observed at 1.0 μM Cd but not at lower and higher concentrations (Wold et al., 2017) (Wold et al 2017). Here, we ask if the inconsistencies in bradycardia are due to morphological differences or other compensatory mechanisms.

Over the past decades, selenium (Se) has emerged as a potential therapeutic agent to counteract Cd-induced toxicity in various animal organs and tissues, including the heart(Chen et al., 2017; Li et al., 2013; R. Liu et al., 2018; S. Liu et al., 2014; Tan et al., 2017). Se is an essential trace element for many eukaryotes, including mammals and fish, primarily found in natural food sources as selenomethionine, selenocysteine, selenium-methylselenocysteine (organic forms), and selenate (inorganic form) (Burk & Hill, 2015; Castellano et al., 2005; Whanger, 2002). Selenite, another inorganic form of Se, is naturally present mainly in phytoplankton and is also added to nutritional supplements (Lei et al., 2022). In its form as selenocysteine, Se is incorporated into proteins to form selenoproteins, which are crucial for maintaining redox balance in cells (Lu & Holmgren, 2009; Reeves & Hoffmann, 2009). At low levels, Se exhibits potent antioxidant activity by upregulating selenoproteins such as glutathione peroxidase (GPx) and thioredoxin reductase (TrxR). These enzymes help eliminate reactive oxygen species (ROS) and suppress oxidative stress-mediated cell damage, a major mechanism underlying Cd-induced toxicity and related to the etiology of several chronic diseases, including CVD (Cuypers et al., 2010; Forman & Zhang, 2021; J. Liu et al., 2009; Valko et al., 2006). However, other mechanisms have been proposed to explain the role of Se in alleviating Cd-induced cell damage. Se can sequester Cd, forming a biologically inert compound, thereby reducing Cd accumulation in cells and tissues. This sequestration has been suggested as the major mechanism of action of Se against Cd toxicity (Zwolak & Zaporowska, 2012). The activation of the Nrf2 pathway, a master regulator of cellular redox homeostasis, has also been proposed as a mechanism by which Se counteracts Cd-induced oxidative stress, thereby mitigating its toxicity (Zhang et al., 2017, 2020). However, Se has one of the narrowest therapeutic windows, with a fine line between its protective and toxic effects. Although experimental evidence has demonstrated the protective role of Se against heart damage, including the cardiotoxicity induced by Cd (Cai et al., 2017; Feng et al., 2022), other observational studies and randomized trials have linked Se to an increased risk of cardiovascular disease even at low concentrations (Alexanian et al., 2014; Al-Mubarak et al., 2021; Flores-Mateo et al., 2006; Rayman et al., 2011). These inconsistent findings highlight the need for additional studies to gain new insights into the role of Se in cardiac health and its beneficial doses.

The overall goal of this study was to determine whether or not prophylactic exposure to Se served to alter Cd-induced alterations to cardiac phenotypes. This was achieved by assessing the presence of pericardial edema and measuring heart rate across various combinations of Cd and Se concentrations in developing zebrafish. These endpoints were measured in control, Cd-exposed, Se-exposed, and Se to Cd transferred zebrafish. In addition, we sought to determine if Se protective effects were dependent on the length of Se pre-exposure. We hypothesized that Cd exposure would lead to concentration dependent pericardial edema and bradycardia, as has been noted in other studies on zebrafish (Cheng et al., 2000; P. Liu, Wang, et al., 2021; Mitovic et al., 2021; Yin et al., 2014). Further, we hypothesized that Se would have cardioprotective effects, but that these effects were likely to be dependent on Se concentration and how long zebrafish were pre-exposed.

## Method

### Subjects

We used a wild-type, outbred, 5D strain of zebrafish. The adult fish were housed under standard laboratory conditions: pH range of 7.2–8.2, conductivity range of 513.8–708.2 μS, nitrate range of 0.0–0.5 ppm, temperature range of 23.6–29.0°C, ammonia range of 0.0–0.4 ppm and a light-dark cycle of 14:10 hours. To generate embryos, we placed female and male adult zebrafish in a spawning tank that separated the sexes with a divider. The divider was then removed the next morning to ensure that embryos were similar in age. All collected embryos were bleached with sodium hypochlorite and then placed in a petri dish containing embryo media (E3) with a maximum of 50 embryos per petri dish and placed in an incubator regulated to maintain a temperature of 28.5 ± 1°C. All water that housed the fish including laboratory and E3 media had Cd that was below the detection limit of < 0.1 μg/L (US EPA Method 200.8). All experiments were done at the University of Miami under protocols #21–194LF and #22–028LF approved by the Institutional Animal Care and Use Committee (IACUC).

### Experimental design

**Figure 1:**
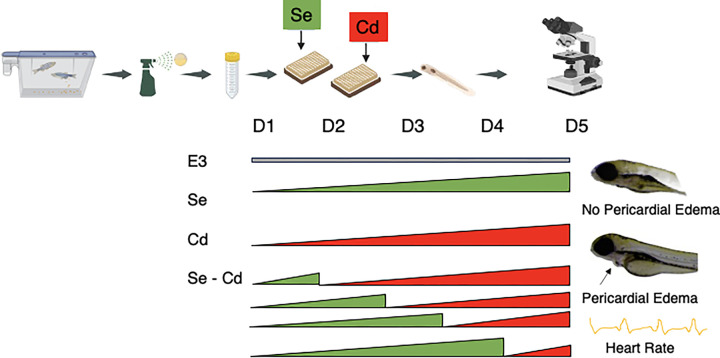
The experimental design included spawning adult zebrafish, collecting the embryos and bleaching them and placing embryos into well-plates by 6 hpf. We placed a subset of the embryos into E3 media (control) and others were placed in Se and Cd dissolved in E3 media. On days 1–4, fish were then transferred to E3, Se, or Cd. On day 5, we assessed the presence (or absence) of pericardial edemas and measured heart rate.

At ≤ 6 hpf, embryos were transferred into well plates containing E3 media (control), while others were placed in Selenious acid (Cas # 7783–00-8, Thermo Scientific) or Cadmium Chloride (Cas # 10108–64-2, Sigma Aldrich) dissolved in E3 media. To assess the therapeutic potential of Se for Cd toxicity, we exposed fish to 0, 10, 50, 100, 150, 200 μg/L of Se and either 0, 2.5 or 5 μg/L of Cd. Fish exposed solely to E3 media served as our control. From days 1–4, fish were transferred to E3, Se, or Cd solutions. If embryos were transferred from Se to Cd, they were rinsed three times in Cd solution prior to transfer. On day 5, we assessed the presence (1) or absence (0) of pericardial edemas by viewing each fish under a Zeiss Discovery v20 microscope (Fig. 1). In a separate experiment, we exposed fish to 2.5 μg/L or 5 μg/L of Cd, 100 μg/L of Se. On day 1, a subset of fish exposed to 100 μg/L of Se were transferred to 2.5 μg/L or 5 μg/L of Cd. Another group exposed to 100 μg/L of Se were transferred to 5 μg/L of Se on day 4. On day 5, we measured the heart rate of the fish by counting the number of heart beats that occurred in 10s while the fish were immobilized on a slide using methylcellulose under a Nikon light microscope (Model 167511). For a subset of fish, we noted the presence or absence of pericardial edemas (Fig. 1).

### Analysis

We used Kruskal-Wallis chi-squared tests to compare fish exposed to Se and Cd across concentrations and days of transfer to identify differences in the presence or absence of pericardial edemas. We fit the chi-squared models using the “kruskal.test” function in the stats package. For Kruskal-Wallis tests that reached statistical significance, we followed up with Dunn’s Multiple Comparisons post-hoc tests using “dunnTest” function in the chisq.posthoc.test package. To identify differences in heart rate across treatment groups, we used a one-way analysis of variance (ANOVA). We fit the ANOVA models using the “aov” function in the base package followed by the “Anova” with type III sums of squares function in the car package for unbalanced ANOVAs (Shaw & Mitchell-Olds, 1993). We examined the residuals to determine if square-root or log transformations were necessary. For ANOVAs that reached statistical significance, we followed with Tukey HSD tests. For comparisons involving heart rate between pairs of groups, we used independent samples t-test (two-tailed). We applied Welch’s correction when data were not equal in variance. Our alpha level was 0.05. We used R for all statistical tests (R Core Team, 2015). Graphs were made using the “base” package in R version 4.2.3 (R Core Team, 2015).

## Results

### Lower concentrations, but not higher concentrations of Se lead to fewer incidences of pericardial edemas

**Figure 2:**
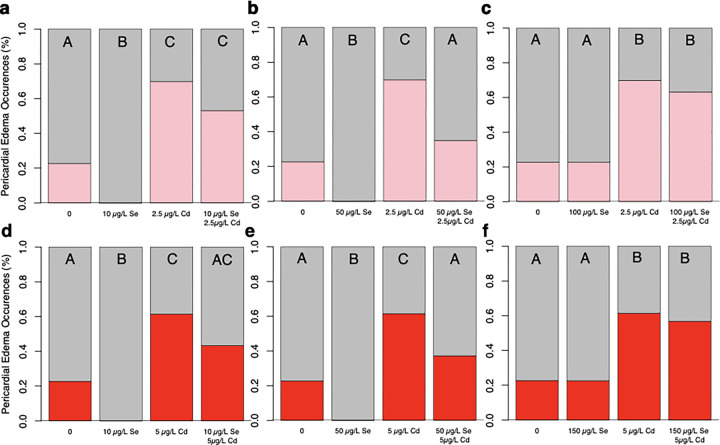
Effects of Se Pre-exposure on Cd-induced pericardial edemas. a) Fish pre-treated with 10 μg/L of Se prior to Cd (*n* = 32) show fewer pericardial edemas compared to fish exposed to 2.5 μg/L of Cd (*n* = 169). However, this difference was not statistically significant. Se-deficient fish (*n* = 167) show fewer pericardial edemas than those treated with 10 μg/L of Se (*n* = 31). b) Fish pre-treated with 50 μg/L of Se prior to Cd (n= 78) exhibit fewer pericardial edemas compared to fish exposed to 2.5 μg/L of Cd (*n* = 169). Control fish (*n* = 167) show fewer pericardial edemas than those exposed 2.5 μg/L of Cd (*n* = 40). c) Fish exposed to 100 μg/L of Se prior to Cd (*n* = 311) and those exposed to 2.5 μg/L of Cd (*n* =169) show similar incidences of pericardial edemas. Control fish (*n* = 167) and those exposed to 100 μg/L of Se (*n* = 159) show similar incidences of pericardial edemas. d) Fish pre-treated with 10 μg/L of Se prior to Cd (*n* = 30) show a similar percentage of pericardial edemas as fish exposed to 5 μg/L of Cd (*n* = 166). Se-deficient fish (*n* = 167) exhibit fewer pericardial edemas than those treated with 10 μg/L of Se (*n* = 31). e) Fish pre-treated with 50 μg/L of Se prior to Cd (*n* = 38) and Se-deficient fish (*n* −167) display fewer pericardial edemas than fish exposed to 5 μg/L of Cd (*n* = 166). Se-deficient fish (*n* = 167) show more incidences of pericardial edemas than fish treated with 50 μg/L of selenium (*n* = 50). f) Fish exposed to 100 μg/L of Se prior to Cd (*n* = 156) and those exposed to 5 μg/L of Cd (*n* = 166) show similar incidences of pericardial edemas. Control fish (*n* = 167) and those exposed to 100 μg/L of Se (*n* = 159) also show similar incidences of pericardial edemas. The letters above each bar show the results of Dunn’s tests. Bars that have the same letter do not significantly differ from each other (*P* > 0.05).

Fish exposed to 2.5 μg/L of Cd exhibited approximately three times more pericardial edemas than unexposed Cd fish (Fig. 2a; *H*(3, *n* = 399) = 101.78, *p* < 0.0001). Among fish exposed to 2.5 μg/L of Cd, those pre-treated with 10 μg/L of Se showed a trend towards 16.7% fewer pericardial edemas, although this difference did not reach statistical significance (Tukey HSD, *p* = 0.08). Fish treated with 10 μg/L of Se displayed fewer pericardial edemas compared to Se-deficient fish. Fish pre-treated with 50 μg/L of Se before exposure to 2.5 μg/L Cd showed less than half the percentage of pericardial edemas compared to those exposed to Cd alone (Fig. 2b). Fish pre-treated with 50 μg/L of Se prior to 2.5 μg/L Cd exposure exhibited a percentage of pericardial edemas similar to control fish. Control fish showed a 22.8% incidence of pericardial edemas, whereas fish exposed to 50 μg/L of Se showed no evidence of pericardial edemas. This difference led to a significant difference in pericardial edemas across the four treatment categories for fish treated with 50 μg/L Se and 2.5 μg/L of Cd (*H*(3, *n* = 416) = 109.26, *p* < 0.0001). Similar results were found for fish pre-treated with 50 μg/L of Se prior to a 5 μg/L Cd exposure (Fig. 2e; *H*(3, *n* = 411) = 79.79, *p* < 0.0001). Se-deficient fish show 22.7% more incidences of pericardial edemas than fish treated with 50 μg/L of selenium (Tukey HSD, *p* < 0.05). Fish exposed to ≥ 100 μg/L of Se showed a similar frequency of pericardial edemas as those exposed to 2.5 μg/L or 5 μg/L of Cd (Fig. 2c, 2f, Supplementary figures). Control fish and fish exposed to ≥ 100 μg/L of Se showed comparable incidences of pericardial edemas. Fish exposed to 2.5 μg/L or 5 μg/L of Cd with or without a ≥ 100 μg/L Se pre-treatment, displayed nearly three times as many incidences of pericardial edemas compared to fish exposed to ≥ 100 μg/L Se and controls. This difference led to a significant difference in pericardial edemas across the four treatment categories for fish treated with 100 μg/L Se and 2.5 μg/L of Cd (*H*(3, *n* = 650) = 128.35, *p* < 0.0001) or 5 μg/L Cd (*H*(3, *n* = 648) = 86.85, *p* < 0.0001).

### Selenium has a narrow therapeutic range

**Figure 3:**
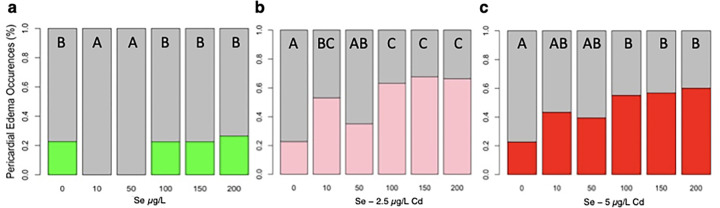
Effects of Se pre-exposure on Cd-induced pericardial edemas. a) Fish exposed to 10 μg/L and 50 μg/L of Se show no incidences of pericardial edemas, whereas Se-deficient and >100 μg/L Se-exposed fish exhibit more pericardial edemas (*n* = 31–167). b) Fish pre-treated with 50 μg/L of Se prior to exposure to 2.5μg/L of Cd show fewer incidences of pericardial edemas compared to other treated groups (*n* = 32–167). c) Fish exposed to Se prior to 5 μg/L of Cd show similar incidences of pericardial edemas (*n* = 30–167). The letters above each bar show the results of Dunn’s tests. Bars that have the same letter do not significantly differ from each other (*P* > 0.05).

Fish exposed to 10 and 50μg/L of Se display no pericardial edemas, whereas 22.7–26.6% of Se-deficient fish and fish exposed to ≥ 100 μg/L of Se show pericardial edemas (Fig. 3a). These differences in the Se-exposed groups led to a significant difference across treatments *H*(5, *n* = 653) = 22.02, *p* = 0.0005. Among fish pre-treated with Se prior to a 2.5 μg/L Cd exposure, 35% of those treated with 50 μg/L of Se showed pericardial edemas, which is 12% more than control fish and 18% fewer than those pre-treated with 10 μg/L of Se (Fig. 3b). Fish pre-treated with higher concentrations of Se, ≥ 100 μg/L of Se, showed 1.8 times more incidences of pericardial edemas than those pre-treated with 50 μg/L of Se. Difference across treatments were significantly different, *H*(5, *n* = 652) = 93.04, *p* < 0.0001. All fish pre-treated with Se prior to a 5 μg/L Cd exposure showed more pericardial edemas than control fish (Fig. 3c). Specifically, 43% and 39% of fish pre-treated with 10 μg/L and 50 μg/L of Se prior to Cd exposure showed pericardial edemas, respectively, whereas those treated with higher concentrations showed 12–20% more pericardial edemas. This difference led to a significant difference across treatments for 5μg/L Cd-exposed pre-treated with Se, *H*(5, *n* = 651) = 58.51, *p* < 0.0001. Dunn’s post-hoc test revealed that all fish pre-treated with Se prior to a 5 μg/L of Cd had statistically similar occurrences of pericardial edemas (*p* > 0.05).

### Longer Se pre-treatments reduce incidences of pericardial edemas in Cd-exposed fish

**Figure 4:**
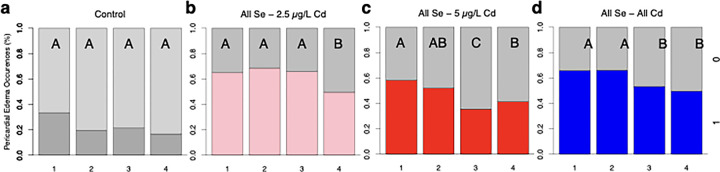
Impact of Se exposure duration on Cd-Induced pericardial edemas. a) Control fish exposed to E3 media show consistent incidences of pericardial edemas across days of transfer (*n* = 41–42). b) Fish pre-exposed to Se for 1–4 days prior to transfer to 2.5 μg/L Cd exhibit reduced incidences of pericardial edemas with longer Se exposure duration (*n* = 120–123). c) Fish pre-exposed to 100 μg/L of Se 1– 4 days prior to being transferred to 5 μg/L of Cd show fewer incidences of pericardial edemas with longer time in Se prior to transfer to Cd (*n* = 156–167). d) Composite figure showing fish pre-exposed to 100 μg/L of Se for 1 – 4 days and then transferred to 2.5 μg/L or 5 μg/L Cd (*n* = 236–246). The letters above each bar show the results of Dunn’s tests. Bars that have the same letter do not significantly differ from each other (*P* > 0.05).

Control fish transferred from well plates on days 1–4 show similar percentages of pericardial edemas. We found that 33% of control fish transferred on 1 dpf show pericardial edemas and 17–21% of control fish transferred on subsequent days experience pericardial edemas (Fig. 4a). These differences did not lead to a significant difference in pericardial edemas across days transferred (*H*(3, *n* = 167) = 3.82, *p* = 0.28). Fish pre-treated with Se for 1–3 days prior to a 2.5 μg/L Cd express at least 15% more pericardial edemas than those pre-treated for 4 days with Se (Fig. 4b). These differences led to a significant difference in pericardial edemas across treatments *H*(3, *n* = 485) = 11.37, *p* = 0.01. Fish pre-treated with Se for 1 and 2 days prior to a 5 μg/L Cd exposure exhibit 58.2% and 51.9% incidence of pericardial edemas, respectively (Fig. 4b). Fish pre-treated with Se for 3 and 4 days show fewer pericardial edemas, with those pre-treated for 3 days showing 22.6% fewer pericardial edemas compared to the 1-day pre-treatment group. These differences led to a significant difference in pericardial edemas across treatments, *H*(3, *n* = 651) = 20.46, *p* < 0.0001. Overall, a Se pre-treatment duration of 3–4 days reduced the occurrence of pericardial edemas by 16.5% compared to those pre-treated for 1–2 days (*H*(3, *n* = 969) = 21.78, *p* < 0.0001).

### Selenium pre-treatment ameliorates Cd-induced bradycardia in 2.5, but not in 5µg/L Cd-exposed fish

**Figure 5:**
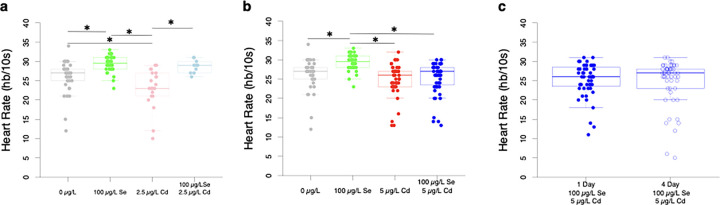
Se and Cd have opposite effects on heart rate. a) Fish exposed to 100 μg/L of Se show elevated heart rates, whereas those exposed to 2.5 μg/L of Cd exhibit lower heart rates compared to control fish (*n* = 10–37). Fish pre-treated with 100 μg/L of Se prior to 2.5 μg/L of Cd exposure and control fish show similar heart rates. b) Fish exposed to 5 μg/L of Cd with and without a 100 μg/L of Se pretreatment have heart rates similar to control fish (*n* = 34–56). c) Fish exposed to 100 μg/L of Se for 1 and 4 days prior to 5 μg/L of Cd exposure display similar heart rates (*n* = 66, n = 48, respectively). Box-and whisker plots overlaid with individual fish represented as circles. *P < 0.05, Tukey HSD.

Se induces tachycardia and Cd’s effect on heart rate is concentration dependent. Fish treated with 100 μg/L of Se and 2.5 μg/L of Cd display bradycardia (Fig. 5a). Fish exposed to 100 μg/L of Se showed 10.7% increase in heart rate compared to control fish. In contrast, fish exposed to 2.5 μg/L of Cd exhibited an 11.8% decrease in heart rate compared to control fish. The 21.3% difference between Se-exposed and Cd-exposed fish was mitigated when fish were pre-treated with 100 μg/L of Se 1 day prior to Cd exposure. Fish exposed to 2.5 μg/L of Cd after 100 μg/L of Se pre-treatment show heart rates similar to control fish. This difference in heart rates across treatments led to a significant difference (*F*(3, 98) = [13.85], *p* < 0.0001). Fish exposed to 5 μg/L of Cd, with or without a pre-treatment with 100 μg/L of Se (M = 24.8, SE = 0.61), display heart rates similar to control fish (M = 26.19, SE = 0.69). Fish exposed to 100 μg/L of Se experience 15.4% and 13.4% faster heart rates than those exposed to 5 μg/L of Cd and fish pre-treated with 100 μg/L of Se for 1 day prior to exposure to 5 μg/L of Cd, respectively. This difference led to a significant difference in heart rate across treatments (F(3, 170) = [10.32], p < 0.0001). Fish pre-treated with 100 μg/L of Se for 1 day and 4 days prior to 5 μg/L of Cd exposure have similar heart rates (Fig. 5c, t(79.35) = 0.94, *p* = 0.35).

### 5 µg/L, but not 2.5 µg/L Cd-exposed fish with pericardial edemas have lower heart rate than those without pericardial edemas.

**Figure 6:**
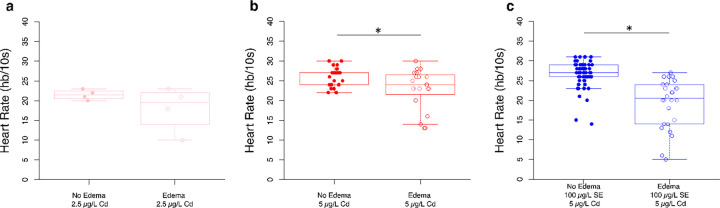
For higher concentrations of Cd, fish with pericardial edemas have lower heart rates. a) Fish exposed to 2.5 μg/L of Cd show similar heart rates independent of the presence of pericardial edemas (*n* = 4 per condition). b) Fish exposed to 5 μg/L of Cd without pericardial edemas have higher heart rates than those with pericardial edemas (*n* = 25, n = 20, respectively). c) Fish exposed to 100 μg/L Se prior to 5 μg/L of Cd without edemas have higher heart rates than those exhibiting pericardial edemas (*n*= 62, *n* = 26, respectively). Individual fish are represented as circles with closed circles representing fish without edemas and open circles representing fish with edemas. Box-and whisker plots overlaid with individual fish represented as circles. *P < 0.05, t-Test.

The influence of pericardial edemas on the heart rate of fish is dependent on the Cd concentration. Fish exposed to 2.5 μg/L of Cd exhibit a 16.3% difference in heart rate depending on the presence of pericardial edemas, but this difference did not reach statistical significance (Fig. 6a, t(3.31) = 1.19, *p* = 0.31). Fish exposed to 5 μg/L of Cd that have edemas show 11.6% fewer beats per min (bpm) than fish without edemas (Fig. 6b, t(25.52) = 2.42, *p* = 0.02). Fish pre-treated with 100 μg/L of Se prior to 5 μg/L of Cd exposure with edemas display 27.8% fewer bpm than those without edemas (Fig. 6c, t(31.01) = 5.79, *p* < 0.001).

## Discussion

The primary objective of this study was to assess whether prophylactic Se exposure could modify Cd-induced changes in cardiac phenotypes. This was achieved by evaluating pericardial edemas and heart rate across various concentration combinations of control, Cd-exposed, Se-exposed, and Se-Cd transferred animals. We also examined if the duration spent in the Se prophylactic treatment influenced Cd toxicity. Our findings confirm that Cd causes pericardial edemas and reduces heart rate, and that Se can attenuate or rescue these phenotypes. However, the potential therapeutic effect of prophylactic Se exposure is both concentration- and time-dependent, with maximal protective effects below 50 μg/L of Se and with longer pre-exposure periods during the developmental period (3–4 days).

As commonly observed in prior studies (Cheng et al., 2000; Mitovic et al., 2021; Yin et al., 2014), exposure to Cd led to pericardial edemas at both 2.5 μg/L and 5.0 μg/L (Fig. 2), and bradycardia at 2.5, but not 5.0 μg/L (Fig. 5). Previous research has documented inconsistent responses in heart rate following developmental Cd exposure, with some showing bradycardia and tachycardia within the same study (Wold et al., 2017). Proposed mechanisms underlying tachycardia include increased apoptosis triggering a compensatory baroreflex response or stress response (Wold et al., 2017). Observations of bradycardia have been attributed to cardiac damage, pericardial edema, altered action potentials, or permanent alterations in sympathetic tone following Cd exposure (Mitovic et al., 2021; Wold et al., 2017). Notably, deviations from the control response could represent lower cardiac performance, which could ultimately impact fitness or increase energetic costs associated with compensatory mechanisms. We found that pericardial edemas tended to correlate with bradycardia at 5.0 μg/L Cd, but not at 2.5 μg/L (Fig. 6), possibly due to the small sample size for the 2.5 μg/L Cd group. More studies pairing heart rate measurements with other cardiovascular metrics can be insightful, as seen in Mitovic et al 2021, where developmental Cd exposure led to increased pericardial edema, reduced heart rate, increased stroke volume, and cardiac output in zebrafish at 96 hpf, while no changes were noted in ejection fraction. Authors in this study suggested that increased stroke volume acted as a compensatory mechanism in response to lower heart rate (Mitovic et al., 2021). As suggested by Mitovic et al 2021, changes in cardiac function are a product of exposure regimes and could be due to a number of factors including changes in genes that encode Na^+^/K^+^ ATPase, myosin heavy chain, L-type Ca^2+^ channels. The effects may be dependent on temperature and the activation of the oxidative stress pathways. In addition to obtaining a better understanding of the mechanisms underlying Cd-induced cardiotoxicity, it is also important to explore therapeutic interventions. While some evidence suggests that interventions that reduce Cd burden may alleviate cardiovascular impacts, more evidence is needed (Diaz et al., 2021).

Our study demonstrated the potential of Se as a therapeutic agent against the adverse effects of Cd on the heart in a concentration-dependent manner. Exposure to Se (100 μg/L for 1 day) rescued Cd-induced heart rate changes (Fig. 5). Reduced heart rate in response to Cd was noted at 2.5 μg/L but not at 5.0 μg/L. While this result was unexpected initially, coupling the heart rate measure with pericardial edemas assessments revealed that fish with edemas showed significantly lower heart rates than those without edemas (Fig. 6). Further, it is important to note that heart rate is just one component of the cardiovascular system that can compensate for Cd-induced impacts. For example, observations of reduced heart rate were accompanied by increased stroke volume in developing zebrafish exposed to 16.7 μM (3 mg/L) Cd for 96 hours, which ultimately led to an increase in cardiac output (Mitovic et al., 2021). Additionally, prior exposure to Se (3–4 days) reduced the occurrence of pericardial edemas caused by Cd at both 2.5 and 5 μg/L, indicating that Se might be used as a preventive treatment for Cd toxicity when Se dosage and time are appropriate (Fig. 4). Moreover, Se (up to 50 μg/L) decreased the pericardial edema rates in our Se-deficient zebrafish controls. Previous studies have highlighted the cardioprotective effects of both organic and inorganic Se compounds, as well as Se nanoparticles (Nano-Se), in alleviating Cd-induced oxidative stress, programmed cell death, and inflammation (Jamall et al., 1989).

However, the same concentration of Se (100 μg/L) that mitigated Cd effects on heart rate also induced pericardial edemas and tachycardia (increased heart rate) in zebrafish, indicating acute Se toxicity (Fig. 5). The effects of Se on heart health are complex and remain controversial. It has been suggested that Se may induce cardiotoxicity in a dose-dependent manner (Flores-Mateo et al., 2006). Nevertheless, there is no consensus about reference levels for Se intake (Medicine et al., 2000; Vinceti et al., 2018) due to little evidence on dose-response relationships between Se and health outcomes. The normal range of Se is based on the Se levels on healthy populations around the world and varies among different regions (Stoffaneller & Morse, 2015; Thomson, 2004; Wasowicz et al., 2003). In the United States, the intake of Se is considered high compared to other countries with an average of 116 μg/daily in people aged 2 years and older (in France, Se intake is 64 ± 14 μg/day in adults) (NIH Office of Dietary Supplements, 2018). This average is higher than the concentration of Se related to cardiac toxicity effects in our study. A recent prospective cohort study in a representative sample of the United States population with non-alcoholic liver disease using The National Health and Nutrition Examination Survey (NHANES)-III (1988–1994) illustrated the non-linear, U-shaped dose-response relationship between dietary Se intake and cardiovascular mortality (Dong et al., 2024). This result indicates that both low and high levels of Se are detrimental to cardiac function.

Our study suggests that more research focused on the investigation of the impact of Se and Cd at different concentrations on cardiac health is needed. In further analyses, we will explore how Se at different concentrations exerts beneficial or toxic effects on the heart at multiple biological levels of organization (molecular, cellular, and physiological, intact animal) using both zebrafish and *in vitro* new approach methods (NAMs) such as primary cells and/or human-induced pluripotent stem cell-derived cardiomyocytes (iPSC-CMs) as models for cardiotoxicity testing. Identifying key gaps in our knowledge about the positive and negative effects of Se on heart health and its therapeutic potential to alleviate Cd-induced cardiotoxicity could help mitigate cardiovascular morbidity. In future studies, it would be useful to overlay the ontogeny of cardiovascular system development with findings present in our student to better understand both mechanisms of Cd cardiotoxicity and the therapeutic potential of Se pre-exposure (Staudt and Stainer 2012). Furthermore, exploration of Cd and Se administered at the same time may have therapeutic implications for populations where ongoing Cd exposure is unavoidable. Finally, the capability of Se to serve as a post-exposure mediation to alleviate negative cardiovascular impacts should also be further explored.
